# Phylogenetic and Evolutionary History of Influenza B Viruses, which Caused a Large Epidemic in 2011–2012, Taiwan

**DOI:** 10.1371/journal.pone.0047179

**Published:** 2012-10-12

**Authors:** Ji-Rong Yang, Yuan-Pin Huang, Feng-Yee Chang, Li-Ching Hsu, Yu-Cheng Lin, Hsiang-Yi Huang, Fu-Ting Wu, Ho-Sheng Wu, Ming-Tsan Liu

**Affiliations:** 1 Centers for Disease Control, Taipei, Taiwan, Republic of China; 2 School of Medical Laboratory Science and Biotechnology, Taipei Medical University, Taipei, Taiwan, Republic of China; The University of Hong Kong, Hong Kong

## Abstract

The annual recurrence of the influenza epidemic is considered to be primarily associated with immune escape due to changes to the virus. In 2011–2012, the influenza B epidemic in Taiwan was unusually large, and influenza B was predominant for a long time. To investigate the genetic dynamics of influenza B viruses during the 2011–2012 epidemic, we analyzed the sequences of 4,386 influenza B viruses collected in Taiwan from 2004 to 2012. The data provided detailed insight into the flux patterns of multiple genotypes. We found that a re-emergent TW08-I virus, which was the major genotype and had co-circulated with the two others, TW08-II and TW08-III, from 2007 to 2009 in Taiwan, successively overtook TW08-II in March and then underwent a lineage switch in July 2011. This lineage switch was followed by the large epidemic in Taiwan. The whole-genome compositions and phylogenetic relationships of the representative viruses of various genotypes were compared to determine the viral evolutionary histories. We demonstrated that the large influenza B epidemic of 2011–2012 was caused by Yamagata lineage TW08-I viruses that were derived from TW04-II viruses in 2004–2005 through genetic drifts without detectable reassortments. The TW08-I viruses isolated in both 2011–2012 and 2007–2009 were antigenically similar, indicating that an influenza B virus have persisted for 5 years in antigenic stasis before causing a large epidemic. The results suggest that in addition to the emergence of new variants with mutations or reassortments, other factors, including the interference of multi-types or lineages of influenza viruses and the accumulation of susceptible hosts, can also affect the scale and time of an influenza B epidemic.

## Introduction

Influenza B and influenza A viruses cause annual epidemics in many regions of the world. Influenza B viruses are not classified into different subtypes based on the HA and NA genes, as influenza A viruses are. However, since 1983 or earlier, isolates of influenza B viruses have been divided into two antigenically and genetically distinct lineages, defined by the reference strains B/Victoria/2/87 (Vic87) and B/Yamagata/16/88 (Yam88) [1], [2]. Viruses of these two lineages have been observed to circulate simultaneously or individually in particular time periods and areas [3]. For example, in the United States, Vic87-lineage influenza B viruses were predominant during the 1980s, but during which time, these viruses were isolated only from sporadic outbreaks in Europe and Asia [2]. During the 1990s, Yam88-lineage influenza B viruses replaced Vic87-lineage viruses and became the predominant strains in the United States. However, both Vic87- and Yam88-lineage influenza B viruses were still detected in eastern Asia during this period. During the 2000–2001 season, Vic87 viruses re-emerged and spread worldwide, and both Vic87 and Yam88 viruses co-circulated in many countries [4]. Thus, the epidemics of influenza B viruses fluctuated periodically and geographically.

The evolution and antigenic drift of influenza B viruses are slower than those of influenza A viruses [5], [6]; nonetheless, various mechanisms of insertion, deletion and reassortment within different lineages increase the genetic diversity of influenza B viruses [5], [7], [8]. Influenza B viruses have experienced relatively frequent segment reassortment events, and reassortments between different lineages have resulted in high genome diversity, and multiple genotypes can co-circulate during a particular time period and in a particular area [3], [5], [9], [10]. Furthermore, it has been shown that the influenza B viruses isolated between 1979 and 2003 belong to at least fourteen genotypes, resulting from genomic reassortments between the descendents of Vic87 and Yam88 viruses [3]. These newly emerging reassortants were associated with unusual influenza B epidemic. For example, in 2002–2003, the worldwide influenza B epidemic was attributed to new reassortant variants [4], [9]. Therefore, analyses of the influenza B gene constellation are an important part of the surveillance of these viruses.

In Taiwan, Yam88- and Vic87-lineage influenza B viruses have circulated/co-circulated during the seasonal or inter-season periods of influenza. Most of the influenza B viruses isolated from 1998 to 2001 belonged to the Yam88 lineage, but some Vic87 viruses were also isolated in March 2001 [11], [12]. In 2002, at least three genotypes of influenza B virus strains were identified, including B/Shanghai/361/2002-like strains (Yam88 lineage), B/Hong Kong/330/01-like strains (Vic87 lineage) and B/Hong Kong/1351/02-like strains (reassortant Vic87 lineage with Yam88 NA) [11], [12]. In the epidemic of 2004–2005, reassortant Vic87 viruses and two genotypes of Yam88 viruses were detected in Taiwan, and the re-emergent reassortant Vic87 viruses also caused the epidemic in 2006–2007 [13], [14]. In the 2011–2012 season, Yam88-lineage viruses were found to uniquely emerge in Taiwan [15], whereas Vic87 lineage viruses circulated in some regions of the world and predominated in China; during this time, influenza A (H3N2) viruses were the major type in Europe and the USA [15]. To track the genomic characteristics and dynamics of the viruses during the large epidemic of 2011–2012 in Taiwan, we analyzed and compared the HA sequences of influenza B viruses collected in Taiwan from 2004 to 2012. The whole genomes of several representative viruses of various genotypes were also included in the analysis. These data allowed the evolutionary processes of influenza B viruses to be elucidated.

## Results

### Laboratory Surveillance of Influenza Viruses in Taiwan

**Figure 1:**
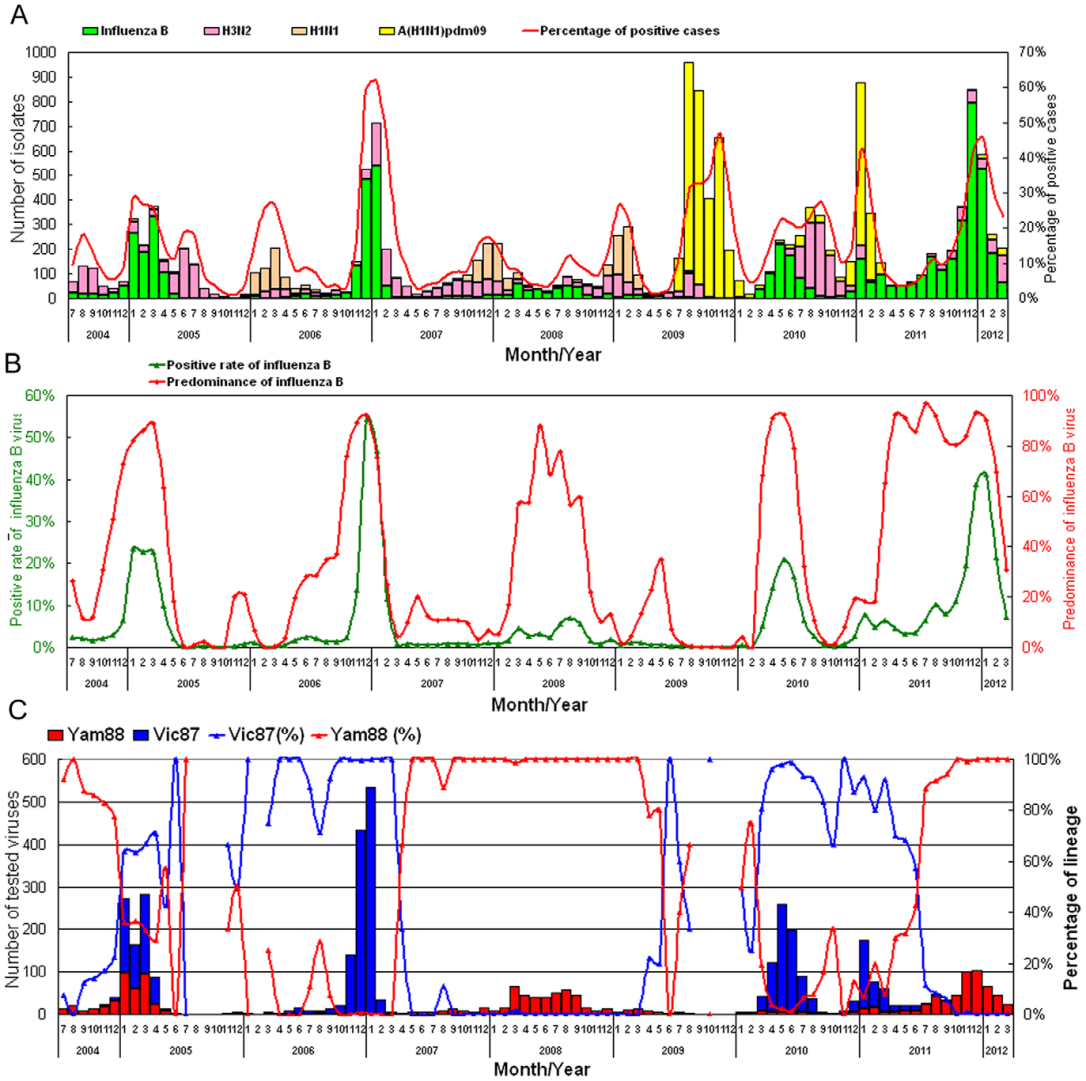
Data from the laboratory-based surveillance network in Taiwan from 2004 to 2012. (A) The monthly distributions of the influenza isolates, including A(H1N1), A(H3N2), A(H1N1)pdm09 and influenza B viruses, are shown as bars. Positive rates of confirmed cases are also shown in the line chart. (B) The dynamic changes in influenza B activity. The monthly positive rates of influenza B represent the percentage of confirmed influenza B cases among all reported and tested cases in a specific month. The predominance of influenza B was calculated by dividing the number of all influenza viruses by the number of confirmed influenza B viruses. (C) The lineage distribution of influenza B viruses. The Victoria (Vic87) and Yamagata (Yam88) lineages isolated from 2004 to 2012 were classified based on their HA genes. The numbers of influenza B viruses analyzed each month are shown as bars, and the monthly ratio of each lineage is shown as a line chart.

The data from the Taiwan influenza surveillance network revealed that the prevalence of influenza subtypes/types varied from season to season. From July 2004 to March 2012, there were six periods predominated by influenza B viruses; three were the influenza seasons of 2004–2005, 2006–2007 and 2011–2012, and the other three were the inter-season periods in 2008, 2010 and 2011 (Fig. 1A, B). Of note, the influenza epidemic of 2011–2012 was larger and longer than previous epidemics caused by influenza B viruses in Taiwan. It was unusual for influenza B viruses to continue to predominate for a long period across the inter-season and seasonal periods. In addition, influenza B viruses replaced influenza A viruses as the major influenza viruses from March 2011 to February 2012. To continuously monitor the genetic characteristics of the evolving influenza B viruses, the Taiwan CDC has regularly performed large-scale analysis of the viral HA sequences [16]. In this study, we analyzed 4,386 (68.2%) of the 6,430 influenza B isolates collected from July 2004 to March 2012. Based on the HA sequences, we genetically classified these influenza B viruses into the Vic87 and Yam88 lineages and their respective sub-lineages. The prevalence of each lineage during this 8-year period, analyzed month by month, is shown in Fig. 1C. Vic87 and Yam88 viruses either co-circulated with each other, or one of the lineages became the predominant type during a period of 1–2 years covering the influenza season and the inter-season period, and then predominance shifted to the other lineage. For example, in 2004–2005, both Vic87 and Yam88 viruses co-circulated. Then, Vic87 viruses were dominant in the 2006–2007 season and in the inter-season of 2010, whereas Yam88 viruses predominated in the inter-season of 2008 and the season of 2011–2012 (Fig. 1B, C). Of note, the unusually large epidemic of 2011–2012 in Taiwan, caused by re-emergent Yam88-lineage viruses, occurred after a lineage switch took place in July 2011 (Fig. 1C).

### Characteristics of Yamagata Lineage Viruses in the 2011–2012 Season in Taiwan

**Figure 2:**
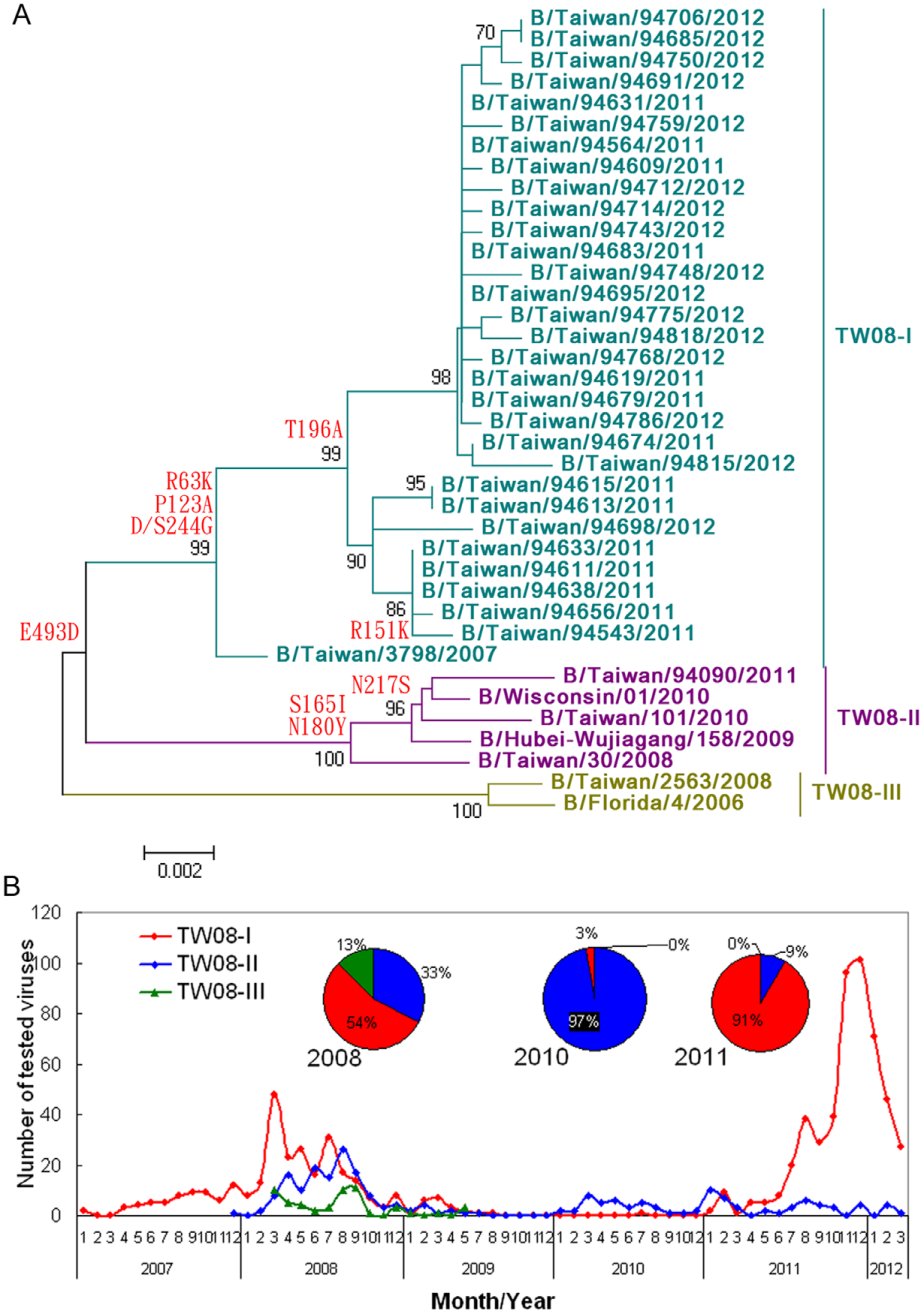
Phylogentic analysis of HA genes and distribution of various genotypes. (A) Phylogenetic topology of the HA genes from multiple genotypes of influenza B viruses from 2007 to 2012 in Taiwan. The phylogenetic trees were constructed using the neighbor-joining method with 1000 bootstrap replications. Branch values of more than 70 are indicated. Based on the phylogenetic analyses and the amino acid signatures of HA genes, the circulating Yam88 viruses were classified into three genotypes, TW08-I, TW08-II, and TW08-III, which are represented by the viruses B/Taiwan/3798/2007, B/Taiwan/30/2008 and B/Taiwan/2563/2008, respectively. The major amino acid signatures of each genotype are as the following: 63K, 123A and 266M for TW08-I; 165I, 180Y, 244D and 266M for TW08-II; and 244S and 266M for TW08-III. (B) The monthly distribution of various genotypes. The monthly numbers of each genotype are shown as a line chart. The inserts indicate the ratios of the three genotypes in 2008, 2010 and 2011.

To determine why the influenza B viruses caused such a large epidemic in 2011–2012 in Taiwan and why these viruses persisted for an unusually long period of time, we first analyzed the complete sequences of the HA genes and compared them with those of viruses that circulated previously. The phylogenetic relationships based on HA showed that most of the viruses from the 2011–2012 season belonged to a large TW08-I genotype (Fig. 2A), which was the major Yam88-lineage genotype and co-circulated with two other genotypes, TW08-II and TW08-III, during 2007–2009 in Taiwan (Fig. 2B). The specific amino acid residues in HA genes of the three genotypes viruses were 63K, 123A and 244G for TW08-I; 165I, 180Y and 244D for TW08-II; as well as 244S and 439E for TW08-III. We further tracked the epidemiologic changes in the TW08-I genotype from 2007–2012. Although the prevalence of TW08-I was the highest (54%) among the co-circulating viruses in 2008, it was replaced by TW08-II in 2010 (Fig. 2B). However, TW08-I re-emerged in 2011. TW08-I viruses first overtook the TW08-II viruses in March 2011, and in July 2011, the TW08-I viruses replaced the Vic87 viruses that had been the predominant viruses circulating in Taiwan since March 2010. Subsequently, the TW08-I viruses caused a large epidemic in 2011–2012 in Taiwan. There were relatively fewer TW08-II isolates in 2011–2012, and the TW08-III genotype was not detected after May 2009 (Fig. 2B). With respect to the antigenicity of these TW08-I viruses, ferret antisera against each of the genotypes were used for the hemagglutination inhibition (HI) assays. Based on the results of HI tests (Table 1), of the 2011–2012 viruses tested using antisera raised against the prototype TW08-I virus B/Taiwan/3798/2007, TW08-II virus B/Taiwan/30/2008 and TW08-III virus B/Taiwan/2563/2008, they all showed good reactivity compared with the titer against the homologous virus. Some of them showed slightly reduced reaction (a 4-fold reduced titer compared with the titer against the homologous virus) with antisera raised against the TW08-I virus B/Taiwan/94748/2012 and TW08-II virus B/Taiwan/101/2010. The results suggested that TW08-I viruses circulating in 2011–2012 were antigenically similar to those in 2007–2009. We also observed that antisera raised against TW08-I viruses B/Taiwan/3798/2007 and B/Taiwan/94748/2012 reacted less with TW08-II reference viruses, resulting in a >4-fold reduction in the HI titer. It indicated that the Yam88 viruses of 2007–2012 have evolved genetically into three genotypes and some isolates in TW08-II seemed to diversify antigenically.

**Table 1:** Antigenic analysis of the Yamagata lineage influenza B viruses by hemagglutination inhibition assay.

viruses	Post-infection ferret sera					Genotype	Collection Date(yy/mm/dd)	PassageHistory*
	TW/3798	TW/30	TW/2563	TW/101	TW/94748			
Reference viruses								
B/Taiwan/3798/2007	**640**	640	1280	160	1280	TW08-I	2007/5/28	C3
B/Taiwan/30/2008	80	**320**	320	320	320	TW08-II	2008/02/22	C3
B/Taiwan/2563/2008	640	640	**1280**	160	640	TW08-III	2008/04/07	C3
B/Taiwan/101/2010	80	320	320	**320**	160	TW08-II	2010/04/10	C3
B/Taiwan/94748/2012	640	1280	1280	160	**1280**	TW08-I	2012/01/14	C3
Test viruses								
B/Taiwan/2140/2009	640	640	1280	160	640	TW08-I	2009/3/19	C3
B/Taiwan/2180/2009	640	640	1280	160	640	TW08-I	2009/3/30	C3
B/Taiwan/94564/2011	640	320	1280	80	640	TW08-I	2011/12/17	C3
B/Taiwan/94786/2012	640	1280	1280	160	640	TW08-I	2012/02/14	C2
B/Taiwan/94543/2011	640	640	1280	160	640	TW08-I	2011/12/09	C3
B/Taiwan/94685/2012	640	640	1280	80	640	TW08-I	2012/01/03	C3
B/Taiwan/94691/2012	640	640	1280	160	640	TW08-I	2012/01/03	C4
B/Taiwan/94619/2011	640	640	1280	160	1280	TW08-I	2011/12/28	C4
B/Taiwan/94712/2012	320	640	1280	80	640	TW08-I	2012/01/05	C3
B/Taiwan/94714/2012	640	640	1280	160	640	TW08-I	2012/01/06	C4
B/Taiwan/94818/2012	640	640	1280	80	640	TW08-I	2012/03/05	C3
B/Taiwan/94609/2011	640	640	1280	160	640	TW08-I	2011/12/24	C3
B/Taiwan/94679/2011	640	640	1280	80	640	TW08-I	2011/12/31	C2
B/Taiwan/94631/2011	320	320	1280	80	640	TW08-I	2011/12/28	C3
B/Taiwan/94698/2012	640	640	1280	80	640	TW08-I	2012/01/04	C3
B/Taiwan/94611/2011	320	640	1280	80	320	TW08-I	2011/12/26	C3
B/Taiwan/94633/2011	640	640	1280	80	320	TW08-I	2011/12/28	C2
B/Taiwan/94656/2011	640	640	1280	160	320	TW08-I	2011/12/29	C3
B/Taiwan/94638/2012	640	640	1280	80	320	TW08-I	2011/12/28	C3

* Passage history of virus was indicated by the number; e.g. viruses of second generation propagated in MDCK cells from clinical specimens were indicated as C2.

### Evolutionary Histories of Yamagata Lineage Viruses in Taiwan

**Figure 3:**
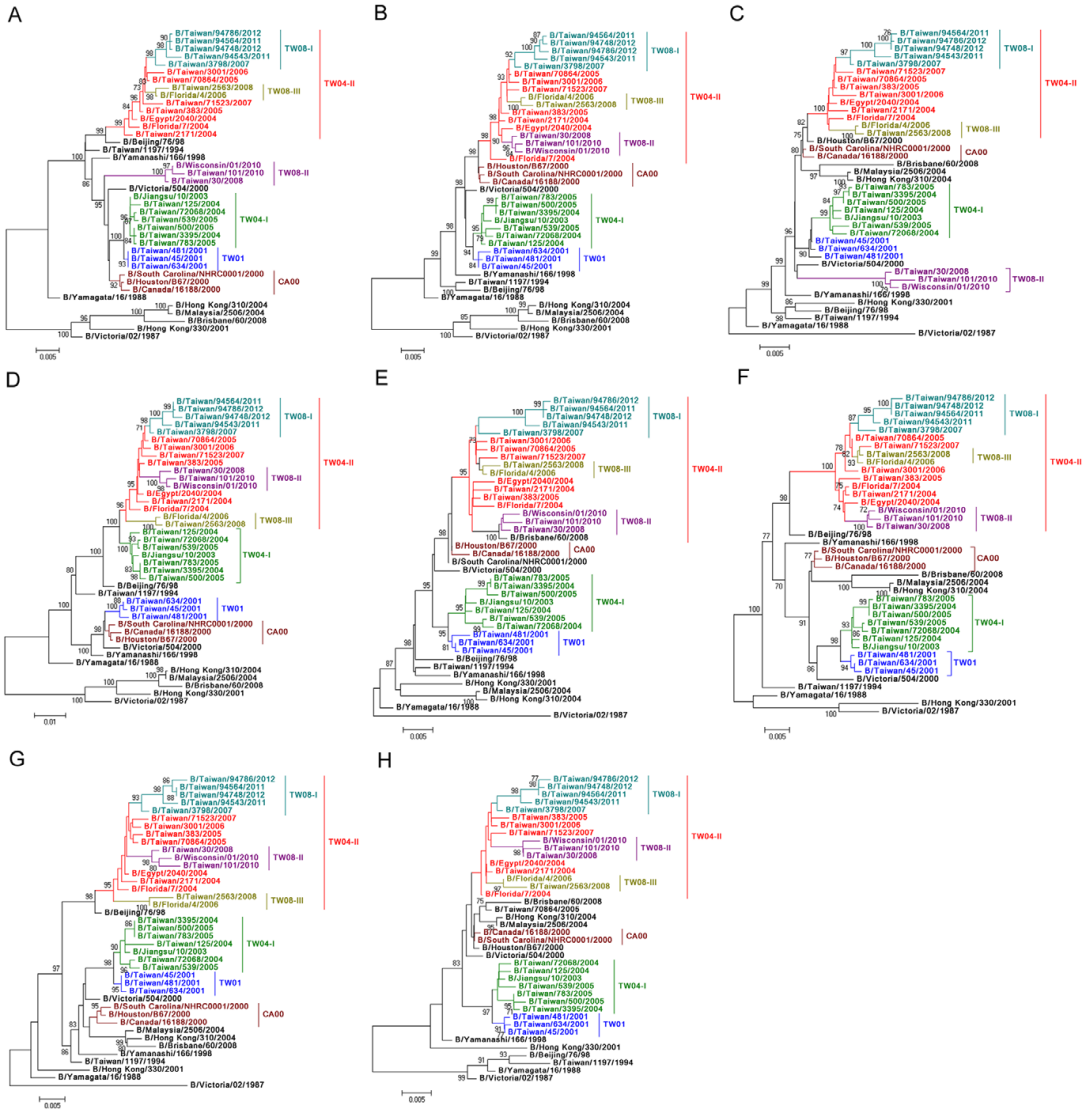
Phylogenetic relationships of multiple genotypes of influenza B viruses from 2007 to 2012 in Taiwan based on the complete sequences of (A) PB1, (B) PB2, (C) PA, (D) HA, (E) NP, (F) NA, (G) MP and (H) NS. The phylogenetic trees were constructed using the neighbor-joining method with 1000 bootstrap replications. Branch values of more than 70 are indicated. The complete genome sequences of the representative TW08-I, TW08-II and TW08-III viruses isolated from 2007 to 2012 and those of viruses that circulated in Taiwan and in other countries from 1994 to 2007 for which sequences were available from NCBI were analyzed and compared. The reference strains B/Victoria/02/1987 and B/Yamagata/16/1988 and the vaccine strains B/Florida/4/2006 and B/Wisconsin/01/2010 were also included in the analyses. The classification of specific evolutionary clades is indicated. The specific amino acid residues in the HA genes of each genotype were as the following: 178E for TW01; 144N, 194V, 247A and 270R for TW04-I; 266M for TW04-II; 63K, 123A and 266M for TW08-I; 165I, 180Y, 244D and 266M for TW08-II; and 244S and 266M for TW08-III.

**Figure 4:**
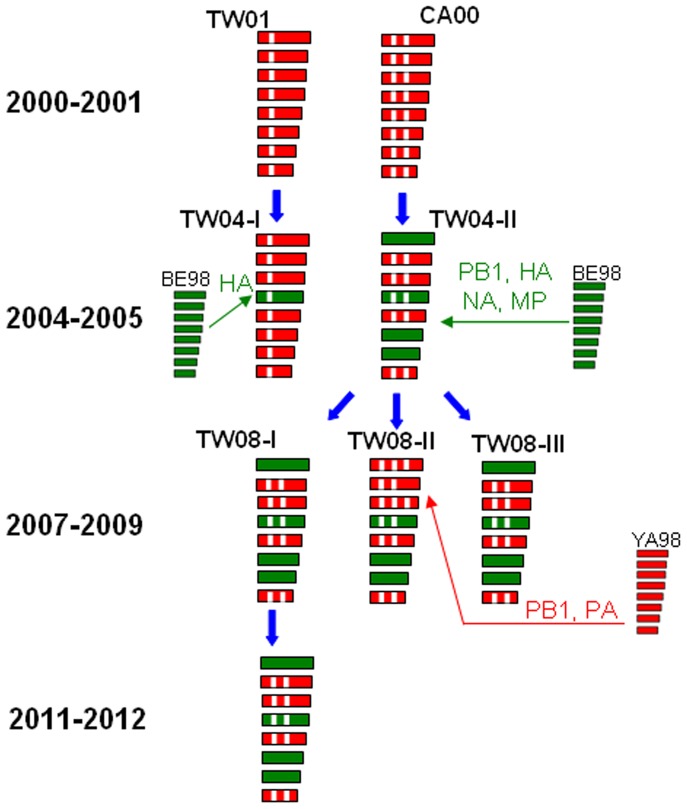
Diagram representing the evolutionary histories of Yamagata-lineage influenza B viruses in Taiwan. The genome compositions of various genotypes are based on the phylogenetic topographies in Fig. 3, and the genotype definitions and representative viruses are described in the legends of Fig. 2 and 3. The gene segments of the viruses are presented in the order of PB1, PB2, PA, HA, NP, NA, M, and NS (from top to bottom). The circulation time period of each genotype is also illustrated. Blue arrows represent possible evolutionary pathways of genotype development. Green arrows represent reassortment between BE98 and existing genotypes. For example, the TW04-II was generated through reassortment between CA00 virus, providing PB2, PA, NP and NS segments and BE98 virus, providing PB1, HA, NA and MP segments. The red arrow indicates the acquisition of segments from YA98 through reassortment. CA00: B/Canada/16188/2000-like viruses; BE98: B/Beijing/76/98-like viruses; YA98: B/Yamanashi/166/98-like viruses.

To understand in detail the genetic composition and molecular evolution of the TW08-I viruses isolated in 2011–2012, whole-genome sequence analyses of the Yam88 lineage viruses that previously circulated in Taiwan were performed. Eight representative viruses isolated during 2007–2012 were selected among the genotypes of TW08-I, TW08-II and TW08-III. In addition, the available complete genomic sequences of viruses that circulated in Taiwan and other countries from 1994 to 2007, together with B/Victoria/02/1987, B/Yamagata/16/1988 and the vaccine strains B/Florida/4/2006 and B/Wisconsin/01/2010 were downloaded from the NCBI database and included in the following phylogenetic analyses. Based on the phylogenetic relationships of the viral genome (Fig. 3A–H), all the eight genome segments of TW08-I and TW08-III viruses belonged to a large earlier TW04-II clade, which was one of the two major evolutionary genotypes of Yam88-lineage viruses in the 2004–2005 influenza season, Taiwan, in their respective phylogenetic trees. Regarding the TW08-II virus, six of the eight segments (PB2, HA, NP, NA, MP and NS) belonged to TW04-II genotype in the respective tree, except for PB1 and PA. The PB1 of TW08-II viruses was more closely related to those of the B/Yamanashi/166/1998-like (YA98), B/Canada/16188/2000-like (CA00) and B/Taiwan/45/2001-like (TW01) viruses, which are older viruses from the Yam88 lineage (Fig. 3A). The PA segments of TW08-II clustered together with those of B/Victoria/504/2000-like viruses and were also located close to that of the YA98 virus (Fig. 3C). These results indicated that these three genotypes, TW08-I, II and III, that co-circulated in Taiwan during 2007–2012 were all derived from TW04-II, and reassortment events were involved in the generation of TW08-II viruses. We also extensively tracked the evolutionary histories of the TW04-I and TW04-II viruses in 2004–2005. The PB2, PA, NP and NS segments of TW04-II viruses could be traced back to those of the CA00-like viruses, and the other four segments, PB1, HA, NA and MP, were closely related to B/Beijing/76/1998-like (BE98) viruses (Fig. 3A–H). For the TW04-I viruses, seven gene segments (all except HA) could be traced back to TW01-like viruses; the HA segment was more closely related to that of the BE98 virus (Fig. 3A–H). In addition, the phylogenic patterns of the PB2, NP and NS segments were congruent (Fig. 3A, E, H), as were those of NA and MP (Fig. 3E, F), indicating that the evolutionary histories of these particular genome segments of Yam88 viruses from 2004 to 2012 in Taiwan have similar patterns. The possible pathways of the evolutionary events and the origins of each genetic segment are presented in Fig. 4.

The comparison of the putative amino acid substitutions of the 11 proteins from TW08-I, II and III is illustrated in Table 2. The TW08-I viruses from 2011–2012 exhibited ten major drift mutations relative to the viruses from 2008: T457A in PB1; S530L in PA; T196A in HA; T107I and S296R in NA; N45D, P67S and V82M in NB; S59N in M2; and V87I in NS2 (Table 2). Other amino acid signatures of the TW08-II and TW08-III viruses were also indicated (Table 2). The results suggest that the large 2011–2012 epidemic in Taiwan was caused by Yamagata lineage influenza B viruses that were derived from the TW08-I viruses of 2007–2009 and the evolution of the 2011–2012 epidemic viruses was developed by drift mutations in several gene segments, with no significant reassortments.

**Table 2:** Amino acid substitutions of multiple genotypes in Taiwan.

genotype	Amino acid residue (of the gene)																																									
	PB1							PB2	PA			HA							NP	NA									NB					M1	BM2			NS1				NS2
	38	66	357	390	391	457	652	555	485	530	617	63	123	165	180	196	244	493	534	42	107	126	187	249	296	341	464	466	13	45	67	82	84	105	21	59	108	110	114	119	179	87
TW08-I (2011–2012)	H	V	V	R	K	A	K	M	V	L	V	K	A	S	N	A	G	D	V	Q	I	T	K	V	R	D	D	A	P	D	S	M	I	K	M	N	S	N	Q	T	M	I
TW08-I *(2008)	**.**	**.**	**.**	**.**	**.**	T	**.**	**.**	**.**	S	**.**	**.**	**.**	**.**	**.**	T	**.**	**.**	**.**	**.**	T	**.**	**.**	V	S	**.**	**.**	**.**	**.**	N	P	V	**.**	**.**	**.**	S	**.**	**.**	**.**	**.**	**.**	V
TW08-II*	Y	I	I	K	R	T	R	L	I	S	I	R	P	I	Y	T	D	**.**	I	R	T	K	R	I	S	N	N	T	L	N	P	V	V	**.**	**.**	S	L	K	P	I	**.**	V
TW08-III*	**.**	**.**	**.**	**.**	**.**	T	**.**	**.**	**.**	S	**.**	R	P	**.**	**.**	T	S	E	**.**	**.**	T	**.**	**.**	I	S	**.**	**.**	**.**	**.**	N	P	V	**.**	R	V	S	L	**.**	P	**.**	I	V

* Identical amino acids to those of TW08-1 (2011–2012) viruses were indicated by black dots.

## Materials and Methods

### Collection of Clinical Specimens and Virus Isolates

This study was conducted through the national influenza surveillance network in Taiwan, which is coordinated by the Taiwan Centers for Disease Control (CDC) [16], [17]. The network continually monitors the circulation of influenza viruses and routinely collects clinical specimens from outpatients in communities and from hospitalized patients with influenza-like illness. In this study, the specimens were tested by RT-PCR/real-time RT-PCR [18], [19], and viruses were isolated with Madin Darby canine kidney (MDCK) cells. The isolated influenza strains were then sent to the Taiwan CDC for further genetic and antigenic characterization by genomic sequencing and hemagglutinin inhibition assays. From July 2004 to March 2011, the sequences of HA genes for most of influenza B strains isolated in Taiwan were determined (3829 of 3855, 99.3%). The viruses for HA sequence analysis from April 2011 to March 2012 were selected randomly, and the number of analyzed viruses each month was limited up to 100. The monthly sampled percentages from April 2011 to March 2012 were 42%–12.5% with the overall percentage as 21.6% (557/2575). Viral RNA used for molecular identification and phylogenetic analysis was extracted from the supernatants of cultured isolates using QIAamp Viral RNA Mini Kits according to the manufacturer’s instructions (Qiagen, Santa Clara, CA). Automated extraction was also conducted using the MagNA Pure LC extraction system (Roche). For passage histories of viruses examined in this study, most of the viruses used to analyze their phylogenetic relationships as well as amino acid substitutions were propagated by two passages (C3) after the parental strain (C1) isolated from the respective clinical specimens.

### Genetic and Phylogenetic Analyses of Various Genotypes of Influenza B Viruses

The HA sequences of a 660-bp fragment from the 1608 viruses collected from July 2004 to December 2006 and a 1350-bp fragment from 2778 viruses collected from January 2007 to March 2012 were determined routinely on a large scale using conventional RT-PCR, as previously described [16]. Multiple genotypes were defined based on the analyzed phylogenetic topology of the viral HA genes. Because the full genome sequences of the Taiwanese viruses belonging to the TW01, TW04-I and TW04-II genotypes are available in the NCBI database, these sequences were downloaded as references. For viruses of the TW08-I, TW08-II and TW08-III genotypes, a total of eight representative strains isolated from 2007 to 2012 were chosen for full genome sequencing and were included in subsequent comparisons. Multiple sequence alignments, protein translation and phylogenetic analysis were performed based on the nucleotide sequences using MEGA4 and BioEdit (http://www.mbio.ncsu.edu/BioEdit/bioedit.html). Phylogenetic trees were constructed by the neighbor-joining method, and 1,000 bootstrap replications were performed to evaluate the robustness and determine the best-fitting tree for each gene.

### Hemagglutination Inhibition (HI) Assay

Antigenic relationship of the influenza B viruses of different genotypes was analyzed by hemegglutination inhibition assay. Post-immunized ferret sera were diluted 1∶4 with Receptor Destroying Enzyme (RDE; Denka Seiken, Japan) and incubated at 37°C overnight. Thereafter, the enzyme was inactivated by heat treatment (56°C for 30 minutes). Serial two-fold dilutions of sera were prepared in 96-well microtiter plates, followed by addition of 25 µL/well of the standardized antigen (4 hemagglutination units/25 µL). After an incubation period of 15 minutes at room temperature, 50 µL of 0.75% guinea pig’s RBC were added and the plates were incubated at room temperature for 60 minutes. Titer results were indicated as the reciprocal of the highest dilution of serum that inhibited virus-induced hemagglutination. The back titer was also performed and was only accepted when both replicates yielded matching results.

### Sequence Information

The nucleotide sequences of the influenza viruses included in this study have been submitted to GenBank, and their accession numbers are JX266866-JX266956. The accession numbers of viruses analyzed in this study also were listed in Table S1.

### Ethics Statement

According to Articles 46 and 47 of the Communicable Disease Control Act in Taiwan, specimens from patients with suspected infectious diseases should be collected and studied for the purpose of disease control. Influenza is a notifiable disease, and clinical specimens of suspected cases should be collected and tested for influenza virus. The present study was carried out based on the national disease surveillance program, which was established by the Taiwan Centers for Disease Control (CDC). The study did not involve any planned activity that could have been reviewed prospectively by an ethics committee. Based on the Communicable Disease Control Act, we are allowed collect specimens without informed consent from suspected cases. This study was approved for publication by the Taiwan CDC. (Article 46: “For collecting specimens, persons in charge of medical institutions shall be responsible for supervising specimen collection; patients and persons concerned shall not refuse, evade or obstruct specimen collection.” Article 47: “Specimens collected by the preceding Article may be, for the need of disease control, handled and studied.”).

## Discussion

Different types or subtypes of influenza A (H3N2 or H1N1) and B viruses have predominated alternately in the past influenza epidemics in Taiwan (Fig. 1A). Taiwan has experienced influenza B epidemics at a 2- to 5-year interval, and the Vic87 and Yam88 lineages can circulate simultaneously or individually during a particular time period. Human immunity and interactions between different types/subtypes of influenza viruses are considered to shape the trends of fluctuating dominance [5], [20], [21]. When there is a large, long-lasting epidemic of influenza A, influenza B will suffer a major population bottleneck, with greatly reduced activity [5]. In Taiwan, we also observed similar epidemiologic patterns for influenza B; its activity was highly limited by different subtypes of influenza A viruses. For example, the large pandemic of influenza A(H1N1)pdm09 hampered the activity of influenza B viruses, resulting in the long-lasting persistence of influenza B viruses at a low level from 2009 to 2010. The absence of influenza B viruses led to an increase in the size of the susceptible population. Furthermore, the influenza vaccines recommended by WHO that were used in Taiwan during the three influenza seasons of 2009–2012 contained a component of only the Victoria lineage (B/Brisbane/60/2008-like viruses), making the vaccine less effective against the Yamagata lineage. These factors may explain why the large Yam88-lineage influenza B epidemic occurred in 2011–2012. The previous studies have suggested that evolution of influenza B virus was highly influenced by reassortment and insertion-deletion, and less governed by antibody selection [7], [22]. In this study, the TW08-1 influenza B viruses circulated in both 2007–2009 and 2011–2012 were antigenically similar (Table 1) and they kept the same constellation of 8 genome segments without detectable reassortment over the five years, indicating that antigenic drifts and genetic reassortment did not involve in emergence of this influenza B epidemic strain. Therefore, the evolution of influenza B viruses was dynamically driven by multiple factors. In this study, both the interactions between influenza A and influenza B viruses of different lineages or genotypes and the size of the susceptible population were shown to determine the time and intensity of influenza B epidemics and shape their dynamic patterns.

In this study, we provided a detailed flux pattern to track the rise and decline of multiple genotypes of influenza B viruses, and also demonstrated the evolutionary processes that a newly emerging or persistent virus became predominant and caused an epidemic. The virus should acquire genetic changes and overtake the existing predominant virus. In a previous study [12], a new genetic reassortant influenza B virus with the HA of the Vic87 lineage and the NA of the Yam88 lineage was shown to have been isolated since 2002 in Taiwan, and this strain co-circulated with two other Yam88-like and Vic-87-like viruses during the same period. However, the reassortant became one of the two major types in the 2004–2005 influenza season and accounted for 73% of isolates from January to April 2005 in Taiwan [12]. In our study, using HA sequences, we could track the epidemiologic changes in the TW08-I virus from 2007 to 2012. The TW08-I virus, which was derived from the TW04-II virus of 2004–2005, co-circulated with the other two genotypes, TW08-II and TW08-III, during 2008–2009 (Fig. 1 and 2). Interestingly, the TW08-I virus fluctuated during the years following; the TW08-I virus was dominant (54%) among the co-circulating genotypes in 2008 but was replaced by TW08-II in 2010 (Fig. 2B). However, the TW08-I virus re-emerged and successively overtook the TW08-II virus and the other Vic87-like viruses in March and July 2011, respectively. The TW08-I subsequently caused the large epidemic in Taiwan. When comparing the complete genome sequences of TW08-I viruses isolated in 2011–2012 and 2007–2009, there were 1–2 drift-associated amino acid substitutions in each of the PB1, PA, HA, NA, NB, M2 and NS2 segments (Table 2). No significant reassortment was detected among the analyzed TW08-I isolates. The T196A substitution of HA proteins is the only difference between the TW08-I viruses of 2007–2009 and 2011–2012 (Table 2). The T196A substitution was not proposed to locate at antigenic site. This is consistent with the data of HI tests (Table 1) that the TW08-I viruses in 2011–2012 were antigenically similar to those in 2007–2009. The substitutions, K63R, A123P, S165I, N180Y and G244D, and K63R, A123P, G244S and G493E were detected respectively in the other two genotypes, TW08-II and TW08-III, comparied with those of TW08-I viruses. Among these substitutions, S165I and N180Y in TW08-II were reported to locate at antigenic sites [23]. The data is consistent with the data of HI test (Table 1) that the antisera raised against TW08-I viruses reacted less with TW08-II viruses, suggesting that some isolates in TW08-II seemed to diversify antigenically. Based on the data of antigenic similarity between the TW08-I viruses isolated in 2007–2009 and 2011–2012, it is proposed that the large epidemic in 2011–2012 in Taiwan was caused by TW08-I viruses that had circulated persistently in antigenic stasis for 5 years.

The phylogenetic patterns of influenza B viruses usually have longer side branches, which are likely to develop into various lineages or genotypes, than the phylogenetic patterns of influenza A (H3N2) viruses [20]. Influenza B viruses diverged into two major lineages, Vic87 and Yam88, in the 1970s, and the Yam88 lineage continued to develop into multiple sub-lineages [2], [3], [5], [24]. The divergent evolution of influenza B viruses, which resulted in the co-circulation of multiple descendants, provides the opportunity for viruses to exchange gene segments between different lineages and genotypes, thus increasing the genetic diversity of the viruses. The frequent and complex reassortment events of influenza B viruses have been reported based on the incongruent phylogenetic patterns of the viral gene segments [5]. Of note, the evolutionary processes of some viral gene segments were not linear–these segments would disappear from the population, and a novel segment would then appear and emerge. For example, the PA, NP, NA, M and NS segments of Vic87 lineage were detected only before 1996, 1995, 2002, 1996 and 2000, respectively, whereas the novel NS segment, which originated in 1984, entered the population after 1997 [3], [24], [25]. In this study, we also found that the TW08-II virus had acquired PB1 and PA segments from ancient YA98-like strains and these two segments did not have an evolutionary linkage with current isolates, indicating that some minor and persistently circulating lineages/genotypes of influenza B viruses existed and that they maintained and increased the diversity of the gene pools available for future epidemic strains. These characteristics of influenza B highlight the importance of enhanced surveillance and large-scale sequence analyses of these viruses.

In Taiwan, 3 of the 8 winter influenza epidemics from 2004–2005 to 2011–2012 were caused by influenza B viruses, indicating that influenza B accounts for a significant proportion of influenza burden (Fig. 1). Among the 3 influenza B epidemics, viruses in the seasons of 2004–2005 and 2011–2012 were different lineage from that contained in the vaccine. Furthermore, it was reported that the vaccine efficacy for influenza B was highly reduced when the circulating epidemic lineage was not matched with vaccine lineage [26], [27]. Based on the burden of influenza B, vaccine-mismatch strains and the limited cross-protection between the two influenza B lineages, the developed quadrivalent influenza vaccine that includes H1N1, H3N2 and two lineages of influenza B viruses could be expected to increase the effectiveness of vaccine and reduce influenza-related morbidities and mortalities.

## Supporting Information

Table S1 **The accession numbers of sequences used in the study.**
(XLS)
